# Culturally and trauma-informed music therapy: approaches for school settings illustrated through descriptive cases from Norway, the United States and South Africa

**DOI:** 10.3389/fpsyt.2025.1736048

**Published:** 2026-01-12

**Authors:** Kathleen M. Murphy, Viggo Krüger, Karyn Stuart-Röhm, Colton Thorn, Sarah Berry

**Affiliations:** 1 Music Department, State University of New York at New Paltz, New Paltz, NY, United States; 2 The Grieg Academy Music Therapy Research Center, University of Bergen, Bergen, Norway; 3 Department of Music, Faculty of Humanities, University of Pretoria, Pretoria, South Africa

**Keywords:** participation, school settings, trauma-informed practice, under-resourced youth, music therapy, community-based

## Abstract

**Introduction:**

Music-based wellness programs have emerged as promising approaches for supporting youth development and psychosocial wellbeing in school settings, particularly for adolescents exposed to trauma. This paper examines how music therapy and music-based wellness initiatives can promote emotional expression, identity formation, and peer connection across diverse sociocultural contexts.

**Methods:**

We constructed three case examples drawn from observational data collected in school- and hospital-based wellness programs in Norway, the United States, and South Africa. The cases were analyzed through the lenses of sociocultural learning theory and trauma-informed educational practice to explore how these frameworks informed music-based interventions with adolescents.

**Results:**

Across all three settings, co-created music experiences facilitated youth voice, emotional expression, and social connection. Collaborative, trauma-informed approaches minimized power differentials between therapists and students, fostering environments of safety, trust, and cultural responsiveness. These conditions supported student empowerment and agency, despite differing structural and sociocultural challenges across contexts.

**Discussion:**

The findings align with existing research on music therapy with trauma-exposed youth, highlighting the importance of co-creation, collaboration, and culturally responsive practice. Music-based wellness programs grounded in sociocultural and trauma-informed frameworks may offer accessible and effective supports for adolescents in school settings. Further research is needed to examine strategies for integrating music therapy and music-based wellness initiatives into school systems to expand access and sustainability.

## 1 Introduction

Adolescence represents a critical developmental period characterized by complex emotional, cognitive, and social transitions (1, 2). For youth experiencing structural marginalization, forced migration, or familial instability, adolescence may be further complicated by exposure to trauma, which can significantly impact capacity for learning, relational engagement, and emotional regulation within educational settings. Uncertainty and systemic barriers in family or school contexts, forced displacement during early childhood, and migration-related trauma all negatively impact an adolescent’s cognitive development, emotional wellbeing, ability to engage meaningfully with others in school settings, and aspirations for the future (3–5). These researchers suggested the need for trauma-informed educational practices that address the complex realities faced by under-resourced and displaced youth.

Recently, there has been growing recognition among educators, policymakers, and mental health professionals of the need to address not only academic achievement but also the psychological and emotional well-being of students (e.g, 6). This has led to the emergence of trauma-informed and culturally responsive pedagogical frameworks aimed at fostering inclusive and supportive learning environments. The music-based wellness programs described in the literature incorporate an interdisciplinary theoretical framework that integrates sociocultural learning theory (7, 8) with principles of trauma-informed care (9, 10).

From a sociocultural perspective, learning is fundamentally a relational and culturally mediated process. Knowledge is co-constructed through social interaction, with tools such as language, symbols, and artistic practices playing a central role in meaning-making (8). Music, as a deeply cultural and embodied medium, becomes a powerful site for this kind of mediated learning. It facilitates not only skill development but also identity expression, emotional exploration, and peer connection—key developmental needs during adolescence (11, 12).

The lens of trauma-informed care complements the sociocultural approach by emphasizing safety, trust, empowerment, and relational attunement in educational and therapeutic settings. Trauma, whether acute, chronic, or systemic—can disrupt emotional regulation, self-concept, and a sense of agency, especially for youth (13, 14). Trauma-informed practice acknowledges these impacts and promotes environments where students feel secure, seen, and supported. Practitioners combining sociocultural learning theories with trauma-informed care underscore the importance of participatory, choice-driven, and culturally grounded experiences, such as collaborative music-making, that can restore agency, strengthen relationships, and scaffold healing (9).

Music therapy projects in schools are increasingly recognized for their capacity to enhance adolescents’ wellness through promotion of emotional regulation, social connection, and stress reduction (15). Swanson (12) gives a detailed account of how specific elements of music can influence physiological, neurological, and psychological systems which prime students for learning and social engagement. She stresses the importance of ritual, such as the use of greeting songs and familiar routines to facilitate social skill development. Heynen et al. (16) conducted a randomized mixed methods study which examined the efficacy of a school-based group music therapy program for refuge children and adolescents. Interview results suggest that this program strengthened the process of social connectedness leading to a sense of belonging. Further there was a statistically significant decrease in negative affect among participants. Similarly, dos Santos (17) engaged adolescents with a history of aggressive behaviors in group music therapy sessions using a humanistic, collaborative approach to create music experiences. Participants’ reported feelings of safety and belonging in the group setting both of which can provide opportunities for social connection and stress reduction. These two exemplary studies underscore music therapy’s value as a holistic, evidence-based strategy for cultivating wellness in educational environments.

## 2 Case examples of music wellness projects in school settings

We were interested in learning how sociocultural and trauma-informed frameworks informed music therapy practices in school-based wellness programs for adolescents in each of our locations. Therefore, we chose to report our reflections of student responses to the music therapy programs in the form of case vignettes. Descriptive, observational case vignettes are the preferred method for describing and evaluating “real-life” clinical work (18). They provide a connection between randomized controlled trials (RCTs) and evidence that comes directly from a clinician’s work (18, 19). Further, they open the door to more rigorous evaluation of clinical work by identifying areas for future research. The case vignettes included have been constructed based on the author’s observations of how students and music therapists co-constructed musical goals engaged in culturally relevant practices, and created spaces for emotional expression and peer support in three international settings; Norway, the United States and South Africa. They highlight how sociocultural scaffolding and trauma-informed facilitation worked together to foster creative confidence, interpersonal connection, and social-emotional growth.

## 3 Case vignettes

### 3.1 Case example from Norway and a reflection

The *Lyden av Oss* (“The Sound of Us”) music workshop was implemented as a twelve-week afterschool initiative in a culturally diverse lower secondary school in Norway. Each week, sessions were held with small groups of 6–8 students. The activities were student-driven, encouraging participants to take the lead in creating, arranging, and performing music. The program emphasized creativity, collaboration, and self-expression, culminating in live performances where students showcased their work to peers, teachers, and the wider community. Recruitment was carried out in close collaboration with the school to ensure diverse participation and strong engagement from the student body. In the Norwegian school context, where inclusion, well-being, and student participation are key educational goals (20), this kind of music-based initiative supports both individual and collective development. *Lyden av oss* was designed to offer a trauma-informed, inclusive space for creative engagement and emotional exploration. Sessions were facilitated by a community-based music therapist in collaboration with the school’s social educator. They were held in the school’s music room, which was equipped with digital audio workstations, musical instruments, and recording technology. Each session followed a consistent structure, beginning with grounding techniques such as rhythmic clapping, breathing exercises, and guided drumming, followed by improvisational jam sessions, lyric development, and collaborative beat production using platforms like *GarageBand* and *Soundtrap*. Students were invited to contribute personal and cultural musical elements, which led to the integration of Syrian melodies, Somali rhythms, and Norwegian folk motifs into original compositions. These acts of cultural sharing fostered mutual respect and curiosity, transforming the workshop into a site of intercultural dialogue. As the program progressed, participants developed a strong group identity, culminating in the co-creation of a final song titled *Sterkere Sammen* (“Stronger Together”) and a public showcase attended by peers, educators, and family members. Student reflections revealed increased emotional articulation, creative confidence, and peer connectedness, with several participants describing the experience as their first opportunity to express personal narratives in a supportive group setting.

#### 3.1.1 Reflection

From a theoretical perspective, the case exemplifies the application of sociocultural learning theory, wherein knowledge is constructed through collaborative activity and mediated by culturally relevant tools (7, 8). The music therapist’s facilitative role enabled students to engage in shared meaning-making, positioning music not only as a medium of artistic expression but also as a cognitive and communicative resource. The workshop also operationalized trauma-informed educational principles by establishing predictable routines, offering choices, and responding sensitively to emotional distress. For instance, when a songwriting activity on the theme of “home” triggered a strong emotional reaction in one participant, the facilitator provided space for regulation and later supported the creation of a new composition focused on resilience. Such moments underscore the importance of relational safety and emotional attunement in trauma-informed practice (10, 14). Furthermore, the emphasis on student agency and co-authorship aligns with empowerment-based approaches, allowing participants to reclaim voice and identity through creative production (21). Within the Norwegian educational context, characterized by a commitment to inclusion, egalitarianism, and holistic well-being, the workshop serves as an illustrative example of how music-based activities can be integrated into school settings to support mental health, intercultural competence, and social-emotional development. While the case is not intended to be generalizable, it offers valuable insight into the pedagogical and therapeutic potential of participatory music-making as a modality for fostering resilience and belonging among adolescents in diverse educational environments. As we move to the next case below, the focus shifts to a different school context where musical interaction takes on new meanings for students.

### 3.2 Case example from the United States and a reflection

“A Place to Be” was designed as an after-school music-based wellness program for students attending a culturally diverse high school in an under-resourced urban setting in the northeastern United States. This pilot program was designed to provide a no-barrier, no-experience required entry point for students who wished to engage in a variety of music experiences such as listening, song discussion, composing, improvising, recording, and performing as a means of fostering social interaction and wellness. Our aim was to complement the existing music and fine arts programs, and school-based mental health services by giving students the opportunity for a creative means of self-expression and exploration. The program was facilitated by a board-certified music therapist and a music therapy practicum student who were supervised by music therapy faculty from the local university. A trauma-informed, co-collaborative approach was taken to all sessions.

Students were invited to participate in the program by their music teachers and members of the pupil-personnel staff. Weekly sessions, lasting for approximately 1 hour, were held in the school’s Black Box Theater. Chairs were set up in a circle to create a contained space for the sessions in the middle of the large theater space. Each session followed a similar structure: checking in with students, engaging in music activities as suggested by the therapist in collaboration with the participants, and closure. The closing included a review of what the students were taking away from the session, and what they might like to work on the next week. Music experiences included song discussion, adapted piano, guitar, and ukulele lessons, drumming, improvisation, and music creation.

Attendance was minimal and inconsistent as only one student attended most sessions. Over the course of the 8-week program the student became more engaged in sessions and willing to share more about themself, and the resultant positive effect on their mood. They consistently reported feeling anxious at the beginning of the group and significantly more relaxed by the end, the music therapists facilitating the group noticed an increase in their frustration tolerance, and willingness to try different ways of engaging in music. They also noticed a change in affect and posture as the session went on. The student who participated in the program consistently seemed to benefit in terms of anxiety management and ability to tolerate tasks (such as learning how to finger guitar and ukelele chords) that required attention and practice. They also became more active in suggesting how they wanted to engage in music.

#### 3.2.1 Reflection—challenges in program implementation

We chose the name “A Place to Be” for our program after the first session, in which two students who did not know each other ended up leaving the session together talking to each other. These two students did not belong to any other clubs or sports teams but found a place to belong in our after-school program. It is unclear why the second student stopped attending, but the one student who continued to attend enjoyed the program and seemingly benefitted from participating. Many may consider this program not to be successful due to the low level of student participation. However, we as facilitators, along with the school’s music staff, felt the benefit to this one student made the program a success. Upon reflection we believe that our understanding of the concept “structuring safety” (22, p. 95) allowed us to create a space in which the participant thrived. ‘Structuring safety’ acknowledges that harm is always possible given the power dynamics and environmental conditions that are present. Therefore, Reynolds (22) recommends that therapists strive to create *safe or safe-enough* spaces in which ongoing consent, shared agreements, and collaboration make therapeutic work possible (23). This approach also honored the participant’s voice, as they took the lead in deciding which music activities to engage in and when to stop.

We also considered Vygotsky’s (8) concept of “zone of proximal development” as we reflected on the progress that was made in terms of social-emotional and music skill development (p. 85). The zone of proximal development is “the distance between the actual developmental level as determined by independent problem solving and the level of potential development as through problem solving under adult guidance or in collaboration with more capable peers” (8, p. 86). The music therapists observed what the participant was able to do musically, usually through improvisation. They gradually introduced new ways to play an instrument in a structured way (e.g. teaching how to finger a chord on the guitar or ukelele) or by providing improvisatory examples of how to create music spontaneously. Important to note here, is that any new material that was introduced was influenced by what the participant created, and then new ways of playing or creating the music were gently introduced.

We faced many challenges in setting up this program. There were some legal and funding delays that impacted our start date. New programs in any setting, but especially a large urban school, take a while to establish. Recruitment for a program offered by an outside agency, in this case the local university, is challenging. It takes time to develop relationships with school staff who can encourage their students to participate. Despite those challenges, what worked was taking a trauma-informed approach in which the participant’s voice was honored and guided all decisions made by the therapists. Taken together, the above case vignettes illustrate how music therapy operates not solely as an add-on service but as a flexible mode of communication within school life. The following vignette invites the reader to consider how similar processes of music therapy unfold within a school in a hospital setting.

### 3.3 Case example from South Africa and a reflection

As part of music therapy services offered by a local Music Therapy not-for-profit organization (NPO), weekly music therapy sessions were held at small school within a large local hospital treating patients with Tuberculosis (TB). For almost two years, 20–25 children aged five to 14 participated in the weekly group music therapy sessions: all from under-resourced communities marked by poverty, unemployment, and crime. The hospital spans a large area and includes three larger buildings enclosed by high barbed wire fences that house women, men and children separately, connected to the admin and management buildings by network of roads. Majority of the TB patients hail from communities in which group music and singing is deeply and culturally embedded. Our music therapy sessions aimed to provide a safe, musical, and playful space to support their overall wellbeing, development, and sense of community amidst the isolation. Sessions were informed by the Nordoff-Robbins Creative Music Therapy and Community Music Therapy (CoMT) approaches. The children sat on small chairs laid out in a circle in the large school room. Each week, using a guitar and various percussive and smaller melodic instruments, the group would sing our greeting songs and other favorite songs, play instruments, improvise, dance, and play music games. We adapted a well-known South African sports song called “Feeling hot, hot, hot” into a musical greeting experience where each child expressed how they were feeling that day: *pap* (weak) or strong. Each week, the group sang hello to every child in a manner that reflected that child’s chosen feeling. The energy the children exuded, and the engagement and interaction belied the isolating effects of their illness and clinical environment.

The children were very excited when I proposed forming an impromptu marching band The goal was to foster a sense of cooperation, collaboration, community, and reduce their isolation. During sessions leading up to our Marching Band debut, children spoke energetically about sharing their music with others and ‘showing off’ their musical skills to staff and other patients. They negotiated and agreed on songs to include. On the day of our Marching Band ‘debut’, the children marched through the pedestrian roads linking the men’s, women’s and baby’s wards. They danced and sang their chosen songs complete with improvised vocal and instrumental sections using shakers, drums, and tambourines. Staff and patients from the various wards came out to listen, sing, clap and dance along with the children (from across the barbed wire fences). The energy and excitement were contagious. Back at the children’s ward, we talked about our marching band, about sick people and helping others to feel better. Sessions continued for another few months, before the NGO had to cease services at the hospital due to lack of funding.

#### 3.3.1 Reflection: from isolation to togetherness

The children’s group music therapy process aimed to bring culturally informed and negotiated music-making into the hospital context to support their health, development and wellbeing. This music therapy process reflects the collaborative, safe and empowered stances within both trauma-informed and socio-cultural learning theories. Our weekly greeting song created a setting in which the children acknowledged, mirrored and attuned to each other’s physical and emotional expression. The familiarity and consistency of myself as the music therapist, the music, the instruments and our greeting song afforded a safe space, in which each child was seen, heard, and supported. This greeting song (while very long considering the large number of children) affirmed their agency and self-expression. Their enthusiasm agency, and engagement with me and each other is indicative of a sense of safety and trust. Attuning to the vocal and motor expressions of others fosters empathy (24) and may support a sense of belonging and social and cultural inclusion and connection (25) This sense of togetherness and solidarity was necessary and meaningful considering their isolation, and the potential trauma of separation from their parents, families, and home communities.

Lengthy admissions of up to nine months at this South African hospital disrupts social life, including relationships, schooling, and community connections, leading to increased isolation (26). This can have profound psychological and educational effects on children (27). I listened to the children express how they missed their families, friends, schools, and communities. This Marching Band encourage the children to expand their music-making to outside of the school room and share their music with staff and patients through the fences. This experience embodies the notion of music as a social connector (28) and shifted our musicking towards ecological, participatory and performative (29). The children negotiated and collaborated with each other, with support from me. They chose songs and dances, selected instruments; rehearsed their dance moves and improvised melodic sections; then finally performed their music and themselves for other TB patients. Our conversation after the Marching Band performance offered opportunities for reflection – to acknowledge their resilience and empowerment, and create meaning during traumatic times (30). While the Marching Band was a once-off occurrence, it was powerful community music experience through which these hospitalized children could claim their voice, identity, resilience and community.

## 4 Discussion

A trauma-informed approach to music therapy service delivered was necessitate by the life experiences of the children who were involved in each program. Trauma is considered an ongoing formative experience that impacts a child’s identity (31). These three distinct international cases, informed by this approach, focused on social, collaborative, client-led experiences emphasizing the students’ agency and empowerment. This fostered safety and trust while empowering individuals through collaboration and focusing on their strengths (31) as seen in the following examples.

### 4.1 Safety and trust

Children who experience trauma often have their perception of safety shattered (10). Further, trauma experiences negatively impact relationships that should afford safety and protection (32) leaving children with a diminished sense of trust in others and their environment (14, 33). We intentionally considered how to provide both physical and relational safety into our work choosing to incorporate practices that have been suggested by adolescents (e.g. 17, 34). These practices included allowing enough physical space between participants, collaborating in the creation of creating group ground rules, and using consistent and warm way of welcoming participants into the group each week. Further, incorporating student-led music experiences, encouraging open expression of ideas, and providing a stable and consistent environment supported sense of relational safety and trust.

Children are not typically in a position of power in their interactions with adults in the best of circumstances. Traumatic events such as forced migration, living in under-resourced communities, or having a chronic and potentially life-limiting illness remove a child’s sense of power and control (33) and increases that power differential (10). Equalizing this power-imbalance, when possible, allows for children’s voices to be heard and respected in the decision-making process (35). Students in each program were empowered to make decisions and work collaboratively with peers and the music therapists in the co-creation of music experiences approach fostered supportive peer interactions and allowed for the creation of cultural relevant music-making.

### 4.2 Music as a tool for agency and voice

Each case vignette highlights the students’ active engagement in collaboration and co-creation. The music therapy content was shaped by the students taking the lead, co-authoring songs, improvising, planning, and negotiating their music making and performances. This is a powerful expression of their agency, identity and voice (metaphorically and literally). Trauma can disempower and contribute to emotional dysregulation, and executive functioning and interpersonal difficulties (36, 37). The proffered opportunities for self-expression, acceptance and peer connection promoted the students as active ‘agents of change’ (38) and served to ‘rewrite’ their trauma narratives (39). Shifting from isolated to together, rigid to creative, the co-created music therapy spaces repositioned the students as ‘agents of change’, which promoted their resilience.

Collaborative music-making enabled the students to express and reflect their identities through the music, and supported their capacities for self-reflection, self-regulation, and self-expression. Students reflected on their growth in self-expression, confidence, group connection, frustration tolerance, and shifts in mood. When acknowledged and validated by the group, the students’ self-expressed music fostered opportunities for dialogue and collective reflection. This two-way process of expression and reflection termed “musical witnessing as a self-object” by Bensimon (40, p. 250), can support identity construction, especially in adolescence (41). Furthermore, the development of group identity was highlighted in the cases. Bridging the social and cultural diversity of their individual contexts, the students appeared to forge distinct musical micro-communities of belonging characterized by intercultural learning and respect. Music became a social phenomenon (29) created and shared by the students.

Lastly, our observations reflect current research that suggests adolescents’ participation in music experiences can promote overall wellness. Over the past ten years, researchers have identified both psychological and physiological benefits adolescents may experience as a result of their participation in music-based experiences (e.g. 15). This paper focuses primarily on the psychological benefits afforded the participants in each of these programs. However, the physiological and neurological responses to music engagement that underlie the psychological effects shouldn’t be overlooked. These include various neurological (e.g. relaxation response); neurochemical (e.g. dopamine, serotonin, and oxytocin) and associative (e.g. emotional contagion; episodic memory) responses to music which seem to be the underlying mechanism for these benefits (42, 43).

### 4.3 Implications for practice

The case vignettes presented in this paper underscore the importance of embedding sociocultural awareness, collaboration, and client empowerment into everyday practice across school and hospital settings. For music therapists, this translates into creating environments that prioritize safety, trust, and mutual respect, which are core components of trauma-informed care. Practitioners should adopt consistent routines to foster predictability while also incorporating flexible, student- or patient-led decision-making to support autonomy and creativity. For example, allowing students to choose instruments, compose lyrics, or shape performance structures encourages co-creation and empowers participants to take ownership of their musical experiences. Given that institutional contexts often impose constraints, such as limited resources, time, or varying levels of trauma-informed readiness, music therapists must cultivate adaptability, reflexivity, and advocacy skills. This includes continually reflecting on one’s own assumptions, actively seeking feedback from participants, and advocating for policy and structural changes that support trauma-informed, culturally sensitive, and participatory music therapy practices.

It is important to consider that the trauma-informed care (TIC) perspective we used for guidance often has the potential to over generalize or medicalize individual’s experiences (44). As such, critics of this framework will argue that by emphasizing individual past trauma as a primary lens for understanding behavior, TIC has the potential to pathologize normal responses to adversity and reduce accountability for current actions. TIC can also be criticized through a post-colonial lens for relying on Western definitions of trauma.

### 4.4 Future research

As we reflected on our work, we wondered how each student carried what they learned across social/emotional domains, into their everyday life. Further, we were curious to know if others in the students’ lives noticed any changes in overall mood and behavior. These questions are ripe for exploration and will shape our future research. Currently, most research involving youth across disciplines usually fits into one of 3 categories: 1) research on youth, 2) an analysis of how youth’s problems effect their well-being, and 3) problem-focused studies attempting to determine how to help youth survive (45). Additionally, research studies are often developed by the investigator with little input from study participants. This approach, reinforces a top-down power dynamic that is contrary to trauma-informed approach that we believe created meaningful experiences for the students. Based on our observations across all 3 sites, we would argue that moving forward, researchers should in engage in youth participatory action research (YPAR) (46). YPAR focuses on strengths, attempts to yield solutions, and most importantly involves youth as co-researchers in each step of the research process. It requires researchers to be flexible with willingness to act as a collaborator with the adolescents from study conception to presentation of the final results (47).We recommend interviewing students to learn more about their life experiences, and how they believe engagement in music therapy or music-based wellness programs might be beneficial. Following the tenets of YPAR, interview results can inform future research with adolescents as co-creators of the research process from conception through dissemination.

### 4.5 Limitations of the study

The present report is limited by the subjective position of the authors. Hence, observations and reflections are processed without direct inclusion of student voices. As such, the perspectives of the students are represented indirectly through the authors’ interpretations, which may introduce bias or overlook nuances in the participants’ experiences. Additionally, this report focuses primarily on psychological benefits of music therapy, leaving physiological and neurological responses underexplored. The trauma-informed care framework, while providing valuable guidance, may also risk overgeneralizing or medicalizing individual experiences and may reflect predominantly Western conceptions of trauma. As such, by centering Western expertise and universalizing its approach to music therapy settings TIC can unintentionally repeat colonial power dynamics, depoliticize structural violence, and appropriate cultural practices without addressing the systems that continue to cause trauma (48). Finally, the study’s descriptive, case study design limits the generalizability of findings beyond the specific school and hospital contexts included.

## 5 Conclusion

The trauma-informed approach adopted in these three diverse school settings supported the creation of collaborative and co-created spaces, which promoted client empowerment, agency, and their experiences of safety and trust. This approach allowed the students to bring in and make culturally relevant music strengthening connections between students from diverse cultural backgrounds. This paper contributes to a growing body of literature on arts-based mental health promotion in education as it demonstrates the integration of theory and practice across disciplinary and cultural boundaries (21). It also offers guidance for educators, therapists, and program developers seeking to implement sustainable, inclusive, and responsive wellness initiatives through music in school settings.

## Data Availability

The raw data supporting the conclusions of this article will be made available by the authors, without undue reservation.
